# Spectrochemical approach combined with symptoms data to diagnose fibromyalgia through paper spray ionization mass spectrometry (PSI-MS) and multivariate classification

**DOI:** 10.1038/s41598-023-31565-0

**Published:** 2023-03-22

**Authors:** Marcelo V. S. Alves, Lanaia I. L. Maciel, João O. S. Passos, Camilo L. M. Morais, Marfran C. D. dos Santos, Leomir A. S. Lima, Boniek G. Vaz, Rodrigo Pegado, Kássio M. G. Lima

**Affiliations:** 1 Institute of Chemistry, Biological Chemistry and Chemometrics, Federal University of Rio Grande do Norte, Natal, 59072-970 Brazil; 2 Institute of Chemistry, Federal University of Goiás, Samambaia St., Goiânia, GO 74690-900 Brazil; 3 Health Sciences Center, Federal University of Rio Grande do Norte, Natal, RN 59072-970 Brazil; 4 Federal Institute of Education, Science and Technology of Sertão Pernambucano, Floresta, Brazil; 5 Estácio de Sá Goiás, North Regional, Goiânia, GO 74063-010 Brazil

**Keywords:** Computational models, Cheminformatics, Rheumatic diseases

## Abstract

This study performs a chemical investigation of blood plasma samples from patients with and without fibromyalgia, combined with some of the symptoms and their levels of intensity used in the diagnosis of this disease. The symptoms evaluated were: visual analogue pain scale (VAS); fibromyalgia impact questionnaire (FIQ); Hamilton anxiety rating scale (HAM); Tampa Scale for Kinesiophobia (TAMPA); quality of life Questionnaire—physical and mental health (QL); and Pain Catastrophizing Scale (CAT). Plasma samples were analyzed by paper spray ionization mass spectrometry (PSI-MS). Spectral data were organized into datasets and related to each of the symptoms measured. The datasets were submitted to multivariate classification using supervised models such as principal component analysis with linear discriminant analysis (PCA-LDA), successive projections algorithm with linear discriminant analysis (SPA-LDA), genetic algorithm with linear discriminant analysis (GA-LDA) and their versions with quadratic discriminant analysis (PCA/SPA/GA-QDA) and support vector machines (PCA/SPA/GA-SVM). These algorithm combinations were performed aiming the best class separation. Good discrimination between the controls and fibromyalgia samples were observed using PCA-LDA, where the spectral data associated with the CAT symptom achieved 100% classification sensitivity, and associated with the VAS symptom achieved 100% classification specificity, with both symptoms at the moderate level of intensity. The spectral variable at 579 *m/z* was found to be substantially significant for classification according to the PCA loadings. According to the human metabolites database, this variable can be associated with a LysoPC compound, which comprises a class of metabolites already evidenced in other studies for fibromyalgia diagnosis. This study proposed an investigation of spectral data combined with clinical data to compare the classification ability of different datasets. The good classification results obtained confirm this technique is as a good analytical tool for the detection of fibromyalgia, and provides theoretical support for other studies about fibromyalgia diagnosis.

## Introduction

Fibromyalgia (FM) is a rheumatologic condition characterized by symptoms such as generalized pain, fatigue, memory problems and sleep disorder^1^. It is the second most common rheumatic disorder after osteoarthritis^2^. The prevalence rate of FM in the world population is 2 to 4%, with a proportion of approximately 90% of patients being women. FM may be present concomitantly with other diseases such as rheumatoid arthritis, osteoarthritis and systemic lupus erythematosus^3^.

In 1990, the American College of Rheumatology (ACR) published criteria for classifying fibromyalgia as a necessary diagnostic procedure^4^, receiving academic and social recognition, where the presence of multiple pain points would be the central criterion^5^. In 2010, the ACR published new diagnostic criteria for FM^6^, eliminating tender point exams and promoting the Widespread Pain Index (WPI) with Symptom Severity score (SS), among other conditions. From this update, the ACR identified the need for modification, providing complete self-administration of the use of scales and questionnaires, allowing the administration of results in survey and environments where the presence of a clinical interviewer would be difficult^7^. The most recent review of the criteria, carried out in 2016, proposes the existence of generalized pain in four specific regions of the body; the persistence of symptoms during the last 3 months; WPI index ≥ 7 and SS ≥ 5 or WPI between 4–6 and SS ≥ 9; as well as question the validity of the diagnosis independently of others variables^8^.

Despite there are criteria used for FM diagnosis, their limits are not easy to discern, since FM is an arbitrary disease and disorder with broad definitions of pain and multiple symptoms that are heavily influenced by culture^9^. Thus, although efforts have been made to improve the accuracy of FM diagnosis in recent decades, the disease remains underdiagnosed or underrecognized^10^. The diagnosis of FM is late^11^ and the non-definition of the disease can generate overuse of health resources, as well as the decline in visits to primary care sites for depression or fatigue after diagnosis^12^.

The development of new methodologies that can help in the diagnosis of FM through instrumental analytical techniques is proposed in some studies. Among these studies, it is possible to mention investigations of potential biomarkers for the disease with proteomic analysis using MALDI-TOF mass spectrometry in saliva sample^13^ and metabolomics studies with the analysis of urine samples in Gas Chromatography Mass Spectrometry (GC–MS)^14^ and blood plasma samples with Liquid Chromatography Mass Spectrometry (LC–MS)^15^. Classification studies are also mentioned, such as using Raman and Fourier-Transform Infrared (FTIR) spectroscopy in differentiating blood samples from patients with FM and other rheumatic diseases^16^; and, discrimination between samples from patients with and without FM based on blood plasmas through Attenuated Total Reflection Fourier Transform Infrared (ATR-FTIR) spectroscopy^17^ and Paper Spray Ionization Mass Spectrometry (PSI-MS)^18^.

Among these instrumental techniques, we highlight mass spectrometry (MS) which plays a prominent role in the field of laboratory medicine^19^. MS is one of the richest analytical techniques in terms of information and interpretation of the data obtained, especially with complex samples such as blood^20^. Among the different modes of ionization in mass spectrometry, paper spray ionization (PSI) allows for a wide variety of applications, in which samples are analyzed directly, eliminating the need for extensive sample preparation steps or chromatographic separation^21^. Paper spraying can be performed in an open laboratory, where a sample of dried blood or other biofluid is analyzed directly on the paper by applying a high voltage to the wet paper^22^. PSI-MS has been widely explored in research with biological samples, providing rapid analysis of complex biological specimens such as blood, urine, saliva or even tissue^23^. Another analytical tool present in several clinical studies is the multivariate analysis of spectral data, which allows the extraction of the most significant features^24^ and provides as much chemical relevant information^25^. Classification tools combine very well with the resolving power of MS analysis, providing the possibility of rapid, accurate and less invasive diagnosis^26^.

This study comprises the use of PSI-MS in blood plasma samples from patients with and without fibromyalgia, who at the time of blood collection also participated in interviews about living, or not, with certain symptoms present on FM. Symptoms are characterized as clinical variables obtained from scales and questionnaires, such as visual analog scale of pain (VAS); the fibromyalgia impact questionnaire (FIQ); Hamilton anxiety rating scale (HAM); the quality of life index (QL); The Tampa Kinesiophobia Scale (TAMPA); and the pain catastrophizing scale (CAT). Based on the responses presented by the patients and the correlation with the respective blood plasma samples, the aim of this study was the formation of different sets of spectral data, according to the clinical variables and their levels of intensities (mild, moderate or severe), where each dataset was subjected to multivariate classification in order to distinguish samples with and without the disease. The classification models were built by Principal Component Analysis (PCA), Successive Projections Algorithm (SPA) and Genetic Algorithm (GA) as techniques to reduce data dimensions; combined with Linear Discriminant Analysis (LDA), Quadratic Discriminant Analysis (QDA) and Support Vector Machines (SVM) for discrimination.

This approach using both the patients’ symptoms data and their blood plasma spectral signature can improve the reliability of FM diagnosis through appropriate combinations of algorithms with the clinical variables most pronounced by the patient. This method, together with the use of PSI-MS, shows the innovative nature of this study, solving uncertainty problems often found in fibromyalgia diagnosis. The proposal of a simpler, faster and more reliable diagnostic approach can play a significant role in the management of this disease by the patients and clinical professionals.

The use of PSI-MS and multivariate analysis in blood plasma samples from patients with and without fibromyalgia, separated in different datasets according to the main clinical symptom used for the diagnosis of the disease, was carried out aiming to show the potential of this methodology to discriminate fibromyalgia samples and to evaluate possible implications of the clinical variables used for the diagnosis of the disease.

## Results

This study is characterized by case–control classifications, with blood plasma samples from patients with and without fibromyalgia (control group—CG and fibromyalgia group—FG), where they were organized by characteristic symptoms of the disease and which are evaluated in their diagnosis. For each symptom, the samples were distributed from levels corresponding to the stratification of the clinical variable, according to the responses described by patients on scales and questionnaires at the time of blood collection. The arrangement of information on clinical variables is not presented uniformly (see Table S1 of the Supplementary Information) due to the absence of some answers, or the non-correspondence of patients interviewed with the samples analyzed in the different symptoms. In all, each symptom was analyzed: 161 samples in the VAS (78 controls and 83 cases), 167 samples in the FIQ (81 controls and 86 cases), 163 samples in the HAM (78 controls and 85 cases), 163 samples in the QL (78 controls and 85 cases), 161 samples in TAMPA (78 controls and 83 cases) and 158 samples in CAT (78 controls and 80 cases).

The analyzed datasets involve the stratification of clinical variables, that is the levels of symptoms presented, considering the limitations of samples in control or cases groups. To carry out the classifications, analyzes were also carried out with the gathering of the symptoms levels, enabling comparisons and indications between the datasets. In the Supplementary Information (Table S2) from the worked data sets are observed, considering the non-feasibility of classifications in a dataset with reduced samples in any of the groups (CG or FG), which would not favor the models training and testing.

**Figure 1:**
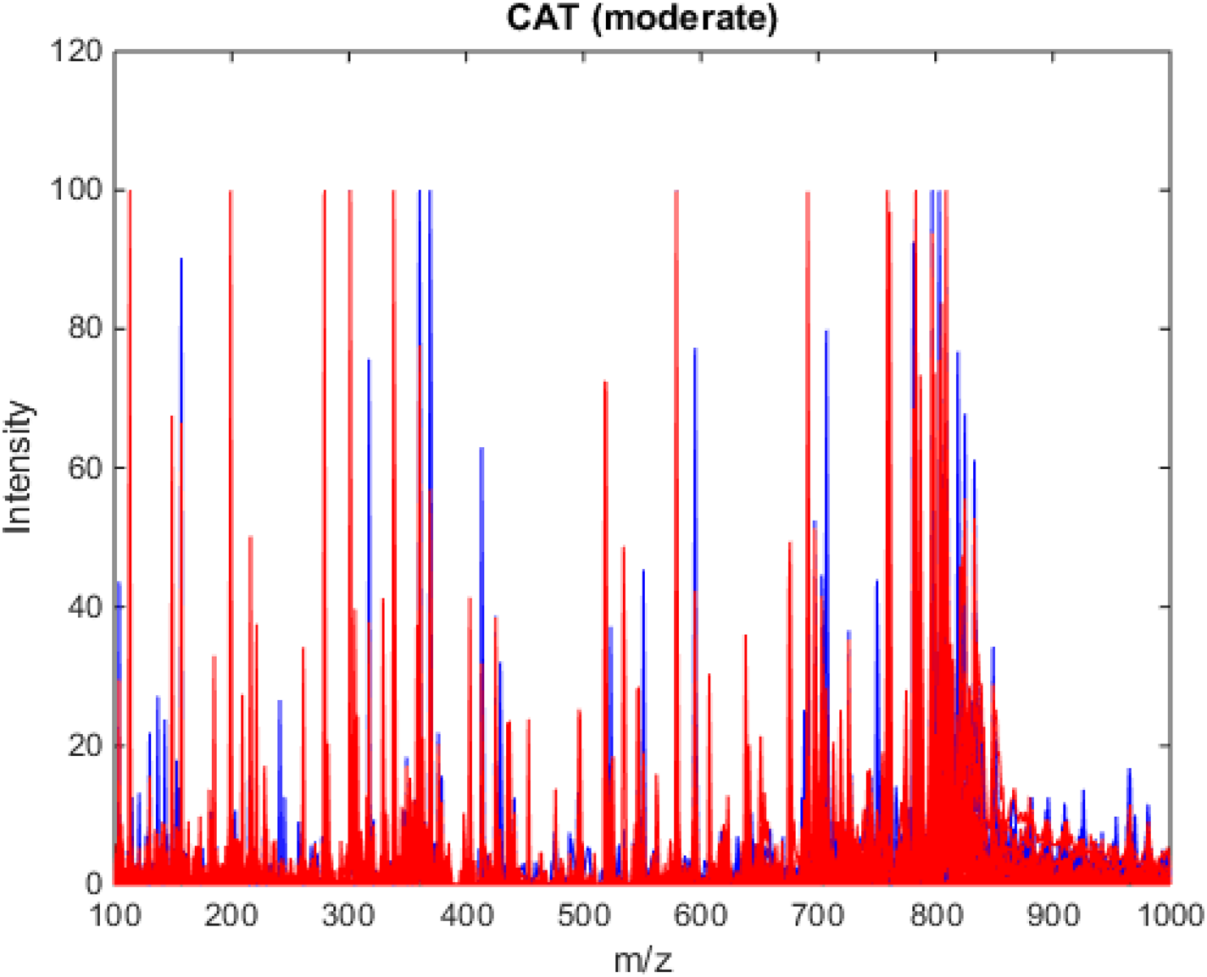
Mass spectra for the moderate CAT symptom, with application of baseline and ROI correction, with samples from the control group (blue) and samples from the fibromyalgia group (red) obtained by PSI-MS. Spectra of the other symptoms can be found in the supplementary information (Fig. S1).

To obtain the best classification results, some initial tests were carried out to improve subsequent processing. The data were pre-processed through the automatic weighted least squares (AWLS) baseline correction^27^. After this first treatment of the raw data, it was necessary to identify information of low-intensity signals in the spectra, to verify if these were relevant for classification. For this, an algorithm to extract regions of interest (ROI) was applied, which consists on the strategy of extracting data included in certain regions and rejecting the remaining data^28^. In this study, we sought to select matrices with *m/z* ratios with intensity above and below 3% at the peak of highest intensity. The tests showed that the matrices with intensity higher than the stipulated percentage value are more favorable for classification. Figure 1 shows the appearance of the total spectra of the analyzed datasets, after application of ROI.

After this first treatment, the pre-processed spectral samples were divided into training (70%) and test (30%) sets using the Kennard–Stone sample selection algorithm. Due to the limited number of samples in some situations, it was decided not to use the validation group to prioritize model tests. Training samples were used to build the classification models, while test samples were used to predict the model performance.

The datasets were submitted to multivariate analysis to distinguish between the control (CG) and fibromyalgia (FG) groups. The classifications were performed with Principal Component Analysis (PCA), the Successive Projections Algorithm (SPA), and the Genetic Algorithm (GA), associated with supervised Linear Discriminant Analysis (LDA), Quadratic Discriminant Analysis (QDA), and Vector Support Machines (SVM), thus nine combinations of algorithms: PCA-LDA, SPA-LDA, GA-LDA, PCA-QDA, SPA-QDA, GA-QDA, PCA-SVM, SPA-SVM and GA-SVM, seeking to identify the combination with the best performance for each dataset.

**Figure 2:**
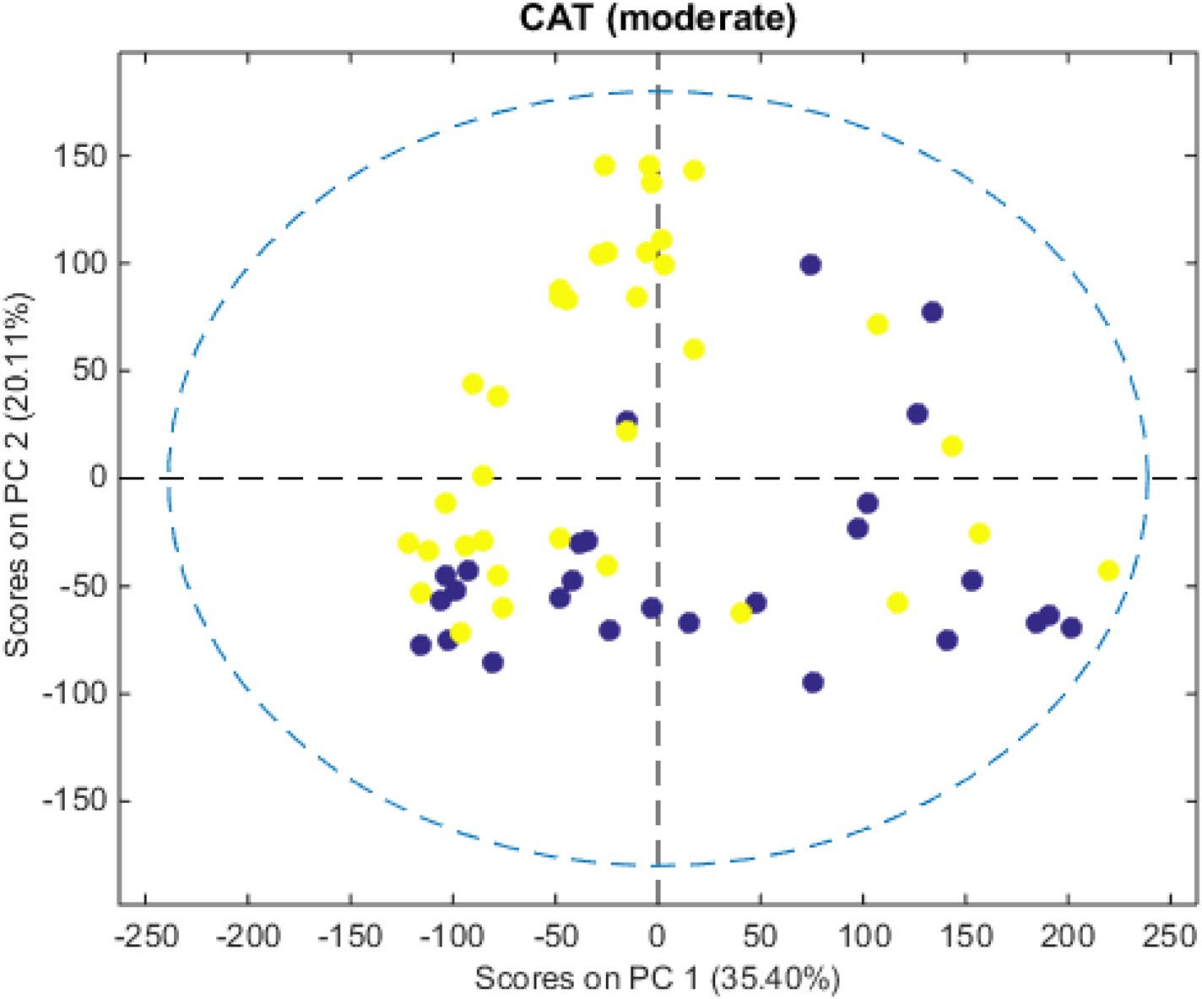
Graphs of PC1 versus PC2 scores for the moderate CAT symptom, with CG (yellow) and FG (blue) samples and confidence ellipses (dashed circles) for the analyzed datasets. The percentage of total variance for each PC is described in parentheses. Graphs of the other symptoms can be found in the supplementary information (Fig. S2).

Before starting the classifications, the PCA was used as an exploratory analysis, resulting in scores graphs for the first and second principal components (PCs). The scores on PC1 and PC2 are shown in Fig. 2. Although PCA can also be considered an unsupervised classification tool, it is observed in all the score charts the absence of patterns in the discrimination of the CG and FG groups. Thus, the use of supervised analyzes was necessary.

The multivariate analyzes were performed following the application of nine different combinations of supervised techniques, seeking the best result in the discrimination of groups for each set of data. The spectral data that presented the best results were those of the mass positive ionization mode. For the PCA analyzes as an algorithm to reduce the dimensions of the data, the first five PCs were used. The SPA and GA algorithms were limited to ten variables as the upper bound in the selection of variables. In the GA, ten generations and ten for the population of individuals in each generation were used. For the SVM algorithms, the Polynomial Base Function (PBF) kernel was used for all spectral datasets. Regarding the results of efficacy in classifying the groups, in each dataset, sensitivity and specificity values were used for the control and fibromyalgia groups. The best results obtained, for each symptom level or sum of levels, are shown in Table 1. The models that best adapted to the spectral data were PCA-LDA, PCA-SVM, GA-LDA and GA-SVM.

**Table 1:** Models with the best performance in each data set, followed by the respective sensitivity (Sens.), specificity (Spec.) and accuracy values.

Clinical variable	Symptom level	Best model	Sens. (%)	Spec. (%)	Classification accuracy (%)
VAS	Mild	PCA-SVM	60	90	75
	Moderate	PCA-LDA	57	100	79
	Mild + moderate	PCA-LDA	68	71	70
FIQ	Moderate	PCA-LDA	50	75	63
	Severe	PCA-LDA	50	100	75
	Moderate + severe	PCA-LDA	73	71	72
HAM	Moderate	PCA-LDA	50	75	63
	Severe	GA-LDA	97	46	72
	Moderate + severe	PCA-SVM	65	90	78
QL	Regular	GA-SVM	77	42	60
TAMPA	Moderate	GA-SVM	79	68	74
	Grave	GA-LDA	71	58	65
	Moderate + severe	PCA-LDA	92	72	82
CAT	Mild	GA-LDA	80	60	70
	Moderate	PCA-LDA	100	75	88
	Mild + moderate	GA-SVM	76	75	76

Except for the QL symptom, moderate intensity was present in all symptoms analyzed with good numbers of samples for most datasets. The moderate level had indices of 100% classification in the control group of the clinical variable VAS (PCA-LDA) and 100% in the cases group (or fibromyalgia) of the clinical variable CAT (PCA-LDA). The moderate level of the TAMPA symptom also showed good distinction between the groups, with an accuracy of 68% and 79% in the CG and FG, respectively, using the GA-SVM model. The severe level of symptoms was analyzed in the variable FIQ, HAM and TAMPA, reaching 100% classification in the FIQ symptom for the control group (PCA-LDA). The mild level was present in the VAS and CAT symptoms and presented a good classification of the groups, where the VAS obtained the best result with the PCA-SVM model and accuracy of 90% and 60% for the CG and FG, respectively, while the CAT symptom reached the best classification index with the GA-LDA model, with accuracy of 60% and 80% in the control and cases groups, respectively.

The results of the sums of the levels were highlighted for most symptoms (FIQ, HAM and TAMPA). Note that for these datasets, all combinations involve the sum of the moderate and severe level: in the moderate + severe combination of the FIQ, the values of separation between CG and FG of the PCA-LDA model are equivalent in 71 and 73% of accuracy, respectively; for the moderate + severe combination of the HAM symptom, the PCA-SVM model has a prevalence in the choice of samples from the control group with 90% accuracy, compared to the cases group with 65% accuracy; the opposite effect is observed in the moderate + severe combination of the TAMPA symptom, with efficacy of 92% in the cases group and 72% in the control group, with the PCA-LDA model. For the other sums of symptom levels, despite not showing the best results within each clinical variable, they showed good classifications of the groups where the mild + moderate combination of VAS had an accuracy of 71% in the control group and 68% in the cases group, with the PCA-LDA, while the mild + moderate combination of the CAT had an accuracy of 75% and 76% in the control and cases groups, with GA-SVM. In the clinical variable QL, the condition evaluated is an intermediate symptom level that achieved a good value in the separation of samples from the cases group (77% accuracy) while the classification of the control group did not have the same success (42% accuracy), using the GA-SVM model.

For the study of the main *m/z* ratios in the differentiation of the groups, the variables selected by the GA models with the best performance in each data set were used, since the SPA models did not present satisfactory processing. This survey showed 261 different variables, considering the triplicate execution of the GA models due to its non-deterministic nature. We considered the values that doubled in amount, reducing the number of samples to 31. Table 2 describes this survey, with the respective *m/z* values. Another variable investigation was performed based on the PC1 loadings of each PCA execution in the data sets. For this, the difference between the averages of the spectra of the control group and the cases group was performed. The analysis focused on the resulting peak regions between these differences, which coincided with the PC1 loadings profiles. Considering the existence of relevant *m/z* ratios in the presence of the disease, negative signal peaks were selected, indicating greater intensity for samples from the cases group. Figure S3 (Supplementary Information) illustrates this operation in all data sets worked. Thus, for the analysis of all levels of symptoms, the variables 301, 311, 325, 338 and 579 were selected. In this way, the selected values (31 by the GA models and 5 by the PCA) were searched on platforms with a database of human metabolomic data such as HMDB^29^ and human lipidomic such as LIPID MAPS^30^.

**Table 2:** Variables selected by the GA models for the levels of symptoms studied.

Symptom	Model	Selected variables (*m/z*)									
VAS (mild)	GA-LDA	22	37	143	180	563	575	**645**	**827**	844	850
		**365**	448	659	681	**707**	726	778	**841**		
		113	**160**	161	189	**336**	**413**	794			
VAS (moderate)	GA-SVM	**133**	349	418	459	551	559	**679**	686		
		92	144	182	326	601	**646**	881			
		17	18	153	**391**	486	**797**	840	**846**	893	
FIQ (moderate)	GA-SVM	26	**160**	196	201	343	627	895			
		91	**365**	518	622	643	776				
		**131**	132	141	**225**	**259**	**389**	**492**	766	**888**	
FIQ (severe)	GA-LDA	261	407	**449**	598	722	732	784	**841**	849	
		**5**	8	**259**	267	**389**	397	434	779		
		200	204	334	**375**	444	599	838	**888**		
HAM (moderate)	GA-SVM	67	122	468	699	700	805				
		159	169	195	231	**255**	302	717	879	887	
		75	147	414	445	477	891				
HAM (severe)	GA-LDA	66	174	386	538	546	**846**	871			
		39	273	**492**	629	702	719	832			
		249	277	330	**391**	421	857				
QL (regular)	GA-SVM	**31**	**72**	155	361	614	708	**797**	886		
		57	263	404	**530**	570	741	823			
		**131**	**225**	**366**	385	485	533	636	729	780	**827**
TAMPA (moderate)	GA-SVM	380	381	383	543	562	649	684	792	877	
		**72**	119	183	394	**478**	584	596	773	803	
		76	**252**	320	387	537	586	624	**707**	869	
TAMPA (severe)	GA-LDA	102	**255**	402	544	602	646	785			
		186	**336**	352	501	535	560	639	**645**		
		100	**375**	432	530	651	712	787	**848**	880	
CAT (mild)	GA-LDA	128	168	393	491	582	683	687	751		
		**5**	101	**252**	350	522	532	618	821		
		187	218	**413**	423	749	814	860			
CAT (moderate)	GA-SVM	55	199	**366**	**478**	597	**679**	786	866	889	
		**31**	69	80	**133**	428	**449**	455	553	872	
		95	121	637	673	695	824	**848**			

Symptoms: *VAS* Visual Analog Scale of Pain, *FIQ* Fibromyalgia Impact Questionnaire, *HAM* Hamilton Anxiety Rating Scale, *QL* Quality of Life index, *TAMPA* The Tampa Kinesiophobia Scale, *CAT* Pain Catastrophizing Scale. Models: *GA-LDA* genetic algorithm with linear discriminant analysis, *GA-SVM* genetic algorithm with support vector machines.

The values in bold represent the variables that occurred in more than one model, totaling 31 different variables.

Based on the HMDB platform, for the variables selected by the GA models, the search for human metabolites followed the configuration established for compounds present in blood samples, with endogenous or exogenous origin, intra or extracellular location, and mean mass and monoisotopic mass values [M + H]+ matching the variable. The relationship of the findings is described in Table S3 of the Supplementary Information, where the following *m/z* ratios and their occurrence in the clinical variables were identified: 645 (mild VAS and severe TAMPA), 679 (moderate VAS and moderate CAT), 707 (mild VAS and moderate TAMPA), 797 (moderate VAS and regular QL), 827 (mild VAS and regular QL), 841 (mild VAS and severe FIQ) and 848 (severe TAMPA and moderate CAT). For this list of findings in the HMDB database, the main classes of metabolites correspond to glycerolipids, steroids and derivatives, glycerophospholipids and sphingolipids. For the variables selected by the PCA, the search followed the variables values and identified correspondence with the value *m/z* = 579 for the LysoPC(22:0/0:0) compound. In the PC1 loadings, this variable is presented by all symptoms, except for VAS (moderate) and CAT (mild).

Using the LIPID MAPS database, with ion configuration in positive mode [M + H] + and mass variation tolerance in *m/z* =  ± 0.1, the variables proposed by the GA model (*m/z* = 131, 133 and 225) corresponded to different types of fatty acids and fatty esters (Table S4). The association of variables with symptoms is described as: variable *m/z* = 131 with symptom QL (regular) and FIQ (moderate); variable *m/z* = 133 with EVA (moderate) and CAT (moderate); and variable *m/z* = 255 with QL (regular) and HAM (moderate). There was no correspondence in the LIPID MAPS database for the variables based on the PC1 loadings.

## Discussion

The study carried out by Wolfe et al.^8^ which resulted in a review of fibromyalgia diagnostic criteria in 2016, demonstrated the comparison of studies using diagnostic criteria recommended by the American Committee on Rheumatology in 1990^4^, 2010^6^ and 2011^7^, seeking to determine the validity, usefulness, potential problems, and necessary modifications to the criteria. Despite proposing some adjustments in the use of the criteria, the study concluded that the criteria had good sensitivity and specificity for all analyzed studies, with an average of 84% and 83%, respectively^8^. Recent studies have also demonstrated good sensitivity and specificity in classifying groups of patients with and without fibromyalgia, using blood plasma samples, among which: Passos et al.^17^ used ATR-FTIR spectroscopy, with results of 89.5% sensitivity and 79% specificity in the classification between controls and fibromyalgia patients using a GA-LDA model; while Alves et al.^18^ used PSI-MS mass spectrometry and reached values of 100% sensitivity and specificity using SPA-LDA and exploratory analyzes with PCA, with small groups of samples (10 controls and 10 fibromyalgia samples). The study presented herein obtained 100% sensitivity and 75% specificity (88% accuracy), with a total number of samples equal to 64 (27 controls and 37 fibromyalgia samples) for the classification performed with the PCA-LDA model in the set of data regarding the moderate CAT symptom. Different sets of data were used, with samples from patients with and without fibromyalgia following the relationships of clinical variables obtained by questionnaires and scales. Blood plasma samples were analyzed by the PSI-MS technique and submitted to multivariate analyses. The distinction between the groups of cases and control samples demonstrated in this study indicates methodologies with different combinations of techniques and good potential in the diagnosis of fibromyalgia.

The use of different datasets, varying the amounts of the controls and cases according to the symptom, demonstrated the good adaptation of the spectral data in most of the developed models. In addition to the CAT symptom, the dataset related to the moderate + severe TAMPA symptom also presented a good classification, where the values of sensitivity and specificity were 92% and 72%, respectively, using the PCA-LDA model with a total number of samples equal to 90 (59 CG samples and 81 FG samples). Overall, these results expand the possibilities of using the PSI-MS methodology coupled with multivariate analysis in the diagnosis of fibromyalgia.

Other results obtained are related to the variables selected in the classifications. The outstanding *m/z* ratios accounted for 36, in a range of 900 different variables, using the GA selection models and an analysis of the loadings on the first PC component with the mean differences between the spectra of the control and cases groups. Among the highlighted variables, there was a correspondence of *m/z* = 10 values with the analyzed compound banks (metabolites and lipids). For the association of human metabolites present in blood plasma, the classes of glycerolipids, steroids and derivatives, glycerophospholipids, sphingolipids and lysophosphatidylcholine were identified. Regarding the classes of lipids, there were references to different fatty acids and their conjugates and fatty esters. Among these relationships, the most relevant is indicated by the association of the variable *m/z* = 579 with the compound LysoPC(22:0/0:0). This *m/z* ratio value was indicated in the analysis of PC1 loadings, being present in most data sets. Another reason that highlights this finding is in a previous study^18^ which also confirms the presence of compounds of the lysophosphatidylcholine class in the samples of the cases group, as well as in studies that present this class of metabolites as a possible biomarker or contributing factor to the fibromyalgia phenotype^31,32^.

Despite associations with lysophosphatidylcholine, the fragmentation analyzes did not confirm this class of metabolites. For values below *m/z* = 100, the equipment used did not provide sufficient resolution for fragmentation. Therefore, further investigations on this class of metabolites should be performed in higher resolution equipment in order to find accurate biomarkers.

Due to the efforts needed to improve the accuracy of fibromyalgia diagnosis through the updated criteria^33^, the combinations of methods demonstrated in this study provides a simple and versatile analysis with minimum sample preparation^34,35^. Future research about fibromyalgia diagnosis can benefit from this approach that uses spectral signatures to identify this disorder within a varied clinical phenotype^36^.

In summary, this study demonstrated good ability to distinguish samples from patients with and without fibromyalgia, using PSI-MS spectrometry and multivariate analysis of blood plasma samples. The differences presented refer to the different sets of data used and the achievement of good classification results with different supervised models and a good number of samples in the CG and FG groups. The use of clinical variables proposes the use of different methodologies in the screening of fibromyalgia, where a symptom more evident by the patient, can refer to a specific diagnostic test, using the models proposed in this study. Another proposition involving clinical variables comprises a combination of diagnostic tests based on the most expressive symptoms, confirming or indicating the presence of the disease. These tests may be viable as there is no need for large volumes of blood for analysis, requiring only a small aliquot of blood plasma equivalent to 10 µL. Despite the advantages and possibilities presented herein, it is important to consider that not all levels of symptoms obtained good accuracy values in the classifications, as well as it was not possible to perform processing in some clinical variables, due to the reduced number of samples. Implications on symptom level and the presence or absence of fibromyalgia were also not considered.

Thus, the methodology presented in this study presents itself as a useful tool in the diagnosis of fibromyalgia, proposing effectiveness, better response time, easy execution and good cost–benefit.

## Experimental

### Plasma samples

This case–control study was carried out following the ethical standards of the Declaration of Helsinki and was approved by the local institutional ethics committee of the University Hospital Onofre Lopes (Federal University of Rio Grande do Norte, Natal, Brazil) under the registration number 2,631,168. Informed consents were obtained from all subjects in this study; and all experimental protocols followed ethics guidelines. For this study, 180 plasma samples were selected, 90 samples from patients in the control group and 90 samples from patients with FM. Data were collected from July 2018 to March 2019 and recruitment was carried out during this period. The study was carried out at the Clinical Epidemiology Laboratory of the Federal University of Rio Grande do Norte, Natal, Brazil. Sociodemographic data (gender, age, education, occupation, status and ethnicity), clinical data (impact of fibromyalgia, anxiety, pain and quality of life) and 10 mL of blood were collected from each patient on the same day.

### Measurements of clinical variables

The anxiety symptom severity can be measured by psychometric tools such as the Hamilton Anxiety Rating Scale (HAM-A)^37^. In the diagnosis of fibromyalgia, the HAM-A scale is used to assess the patient's psychological condition and is related to the HAM-D scale, which assesses depression in individuals^38^. A study carried out by Matza et al.^39^ proposes cut-off points for the HAM-A scale scores, making the results more significant and interpretable for researchers, clinicians and patients, where the intervals are divided into: mild (score from 8 to 14); moderate (score 15 to 23); severe (score equal to 24); and score 7 representing no or minimal presence of anxiety.

The Tampa Kinesiophobia Scale defined as excessive, irrational and debilitating fear of movement, is commonly found in patients with fibromyalgia due to the association between pain/fatigue and movement increasing the patient’s disability^40^. The Tampa kinesiophobia scoring scale follows a specific dynamic of the sum of responses present in a questionnaire with seventeen questions, where the final values can vary from 17 to 68 points, with a mild classification (17 to 34 points), moderate (35 to 40 points) and severe (51 to 68 points)^41^.

General symptoms related to fibromyalgia are also evaluated using the Fibromyalgia Impact Questionnaire (FIQ), which is a self-administered questionnaire designed to assess the components of health status believed to be most affected by FM^42^. The FIQ serves as an effective tool to assess symptoms that impact daily functions such as general well-being, work ability, fatigue, morning fatigue, depression, and others^43^. The FIQ score ranges from 0 to 100, with severity analysis classified as mild effect (score less than 39), moderate effect (score between 39 and 59), and severe effect (score above 59)^44^.

The pain symptom represents one of the most important symptoms in disorders of the musculoskeletal system, being the result of the most common measure in rheumatological diseases such as fibromyalgia^45^. The Visual Analog Scale of pain (VAS) is used to measure pain intensity, where the patient indicates their perception of pain on a 10 cm scale, with the left end labeled “No pain” (0 cm) and the right end “Very strong pain” (10 cm)^46^. Using measurements in millimeters, to improve precision, it is possible to classify pain intensity on the VAS scale as: no pain, with values from 0 to 4 mm; mild pain from 5 to 44 mm; moderate pain from 45 to 74 mm; and severe pain above 75 mm^47^.

The Health Survey Summary Form (SF-36) is a generic instrument to assess health and quality of life (QL), with questions about physical function, bodily pain, general health, vitality, mental health and other perceptions related to health^42^. The Pain Catastrophizing Scale (CAT) is associated with more severe symptoms and a worse adaptation to fibromyalgia, characterized by a pessimistic picture and exaggerated interpretations of pain sensation^48^. Both for the SF-36 questionnaires that measure patients’ quality of life—QL, and for the pain catastrophizing scale—CAT, few studies suggest classifications of intensity in the scores. Considering the total score for QL and CAT and observing the trends in the intensities of the other symptoms, it is possible to propose: the classification of the SF-36 questionnaires, with scores between 0 and 100 and higher indices for better QL, low (0 to 25 points), regular (26 to 50 points), good (51 to 75 points) and excellent (76 to 100 points). For the CAT scale, the score ranges from 0 to 60 points, with the highest score indicating a worse perception of the symptom. Thus, the scale can be classified according to intensity: mild (0 to 20 points), moderate (21 to 40 points) and high (40 to 60 points).

### Samples in the PSI-MS spectrometer

For each selected plasma sample, a 10 µL aliquot was removed and applied to triangular paper (Whatman grade 1, GE Healthcare, USA, 1.5 cm side) and left at room temperature (25 °C) until dry. The triangular papers containing small aliquots of blood were positioned in front of the mass spectrometer (at 4 mm between the tip of the paper and the inlet of the mass spectrometer). The dry paper was held by a metal clip connected to the voltage source of the mass spectrometer, with the tip of the paper at approximately 5 mm. 10 µL of methanol (0.1% formic acid v/v) was applied to the paper to form the electrospray for MS analysis. The analyzes were performed in triplicate measures.

### Instrument parameters

Mass spectra were obtained using a Termo Scientifc LTQ-XL Linear Ion Trap Spectrometer. The optimized parameters were the following: positive ionization mode; capillary temperature 275 °C; capillary voltage of 15 V; 4 kV spray voltage; 50 V tube lens. Mass spectra were acquired using Termo Tune plus software and processed for chemometric analysis using the Xcalibur Analysis package software (version 2.0, Service Version 2, Termo Electron Corporation).

### Computer analysis

Spectral data were processed using the MATLAB R2014b software (MathWorks Inc., Natick, USA) with the PLS Toolbox version 7.8 (Eigenvector Research Inc., Wenatchee, USA). All sets of spectral samples were submitted to pre-processing with automatic weighted least squares (AWLS) baseline correction and application of the regions-of-interest (ROI) algorithm. Spectral samples were divided into training (70%) and test (30%) groups using the Kennard Stone sample selection algorithm. In this division, the training sample set is used to build the model, while the test sample set is used to predict the model performance.

Regarding model construction, Principal Component Analysis (PCA) is a powerful and versatile tool capable of providing an overview of complex multivariate data^49^. With PCA it is possible to reduce a large volume of data in a few principal components (PC) that represent most of the original information^26^. In the Successive Projections Algorithm (SPA), projection operations are used to choose subsets of variables with a small degree of multicollinearity, allowing the detection of specific spectral bands^25^. The Genetic Algorithm (GA) is inspired by natural evolution to become a robust and efficient algorithm at the same time, providing selected variables at each execution of the model, which may indicate a good strategy to explore the features present in the analyzed samples^50^.

PCA as feature selection method as well as SPA and GA as variable selection algorithms can be used in conjunction with supervised classifiers such as: Linear Discriminant Analysis (LDA) and Quadratic Discriminant Analysis (QDA) that aim to find limits that separate groups or samples, with LDA getting linear limits where a straight line divides the variable space into regions, and QDA getting quadratic limits where a quadratic curve divides the variable space^51^. LDA and QDA are based on the calculation of the Mahalanobis distance between the samples, demonstrated in Eqs. (1) and (2) for the classification scores of LDA ($${L}_{ik})$$ and QDA $${(Q}_{ik})$$, respectively^26^:

1 $${L}_{ik}=({\mathbf{x}}_{i}- {\overline{\mathbf{x}} }_{k}{)}^{\mathrm{T}} {\mathbf{C}}_{\mathrm{pooled}}^{-1}\left({\mathbf{x}}_{i}- {\overline{\mathbf{x}} }_{k}\right)-2{\mathrm{log}}_{\mathrm{e}}{\pi }_{k},$$

2 $${Q}_{ik}=({\mathbf{x}}_{i}- {\overline{\mathbf{x}} }_{k}{)}^{\mathbf{T}} {\mathbf{C}}_{k}^{-1}\left({\mathbf{x}}_{i}- {\overline{\mathbf{x}} }_{k}\right)+{\mathrm{log}}_{\mathrm{e}}\left|{\mathbf{C}}_{k}\right|-2{\mathrm{log}}_{\mathrm{e}}{\pi }_{k},$$

where ${\mathbf{x}}_{i}$ is the response vector for sample *i*, ${\overline{\mathbf{x}} }_{k}$ is the mean response vector for class *k*, ${\mathbf{C}}_{\mathrm{pooled}}$ is the pooled covariance matrix, ${\mathbf{C}}_{k}$ is the variance–covariance matrix of class *k*, and ${\pi }_{k}$ is the prior probability of class *k*.

The Support Vector Machines (SVM) algorithm is also combined with PCA, SPA and GA, considering the multidimensionality of data and non-linear limits^52^. For classification with SVM, the calculation follows the equation^53^:

3 $$F\left({\mathbf{x}}_{i}\right)= \mathrm{sign} \left(\sum_{i=1}^{{N}_{SV}}{\alpha }_{\acute{i} }{y}_{i}K\left({\mathbf{x}}_{i}, {\mathbf{z}}_{j}\right)+b\right),$$

where ${\mathbf{x}}_{i}$ and ${\mathbf{z}}_{j}$ are response vectors, ${N}_{SV}$ is the number of support vectors, ${\alpha }_{\acute{i} }$ is the Lagrange multiplier, ${y}_{i}$ is the class membership, $K\left({\mathbf{x}}_{i}, {\mathbf{z}}_{j}\right)$ is the kernel function, and $b$ is the bias parameter.

The classification performance of the algorithms used in this study is evaluated through the sensitivity (SENS) (4), specificity (SPEC) (5) and accuracy (AC) (6), using the set of test samples for each group (cases and controls):

4 $$\mathrm{SENS}=\left(\frac{\mathrm{TP}}{\mathrm{TP}+\mathrm{FN}}\right) \times 100,$$

5 $$\mathrm{SPEC}=\left(\frac{\mathrm{TN}}{\mathrm{TN}+\mathrm{FP}}\right) \times 100,$$

6 $$\mathrm{AC}=\left(\frac{\mathrm{TP}+\mathrm{TN}}{\mathrm{TP}+\mathrm{FP}+\mathrm{TN}+\mathrm{FN}}\right) \times 100,$$

where TP stands for true positive, TN stands for true negative, FP stands for false positive and FN stands for false negative.

## Supplementary Information

Supplementary Information.

## Data Availability

The datasets used and/or analysed during the current study available from the corresponding author on reasonable request.
