# Supplementary material for: Spectrochemical approach combined with symptoms data to diagnose fibromyalgia through paper spray ionization mass spectrometry (PSI-MS) and multivariate classification
Source: Sci Rep. 2023 Mar 22;13:4658. doi: 10.1038/s41598-023-31565-0 (PMC10033633; doi:10.1038/s41598-023-31565-0)
Supplement: Supplementary file 1 — Supplementary Information. [file 41598_2023_31565_MOESM1_ESM.docx]

**Supplementary Information**

**Spectrochemical approach combined with symptoms data to diagnose Fibromyalgia through Paper Spray Ionization Mass Spectrometry (PSI-MS) and multivariate classification**

Marcelo V. S. Alves^1^, Lanaia I. L. Maciel^2^, João O. S. Passos^3^, Camilo L. M. Morais^1^, Marfran C. D. dos Santos^4^, Leomir A. S. Lima^5^, Boniek G. Vaz^2^, Rodrigo Pegado^3^, Kássio M. G. Lima^1*^

^1^Institute of Chemistry, Biological Chemistry and Chemometrics. Federal University of Rio Grande do Norte, Natal 59072-970, Brazil; ^2^Institute of Chemistry, Federal University of Goiás, Samambaia St., Goiânia, GO 74690-900, Brazil .^3^Health Sciences Center. Federal University of Rio Grande do Norte, Natal, RN 59072-970, Brazil; ^4^Federal Institute of Education, Science and Technology of Sertão Pernambucano. ^5^Estácio de Sá Goiás, North Regional, Goiânia, GO 74063-010, Brazil; email:

*kassiolima@gmail.com

**
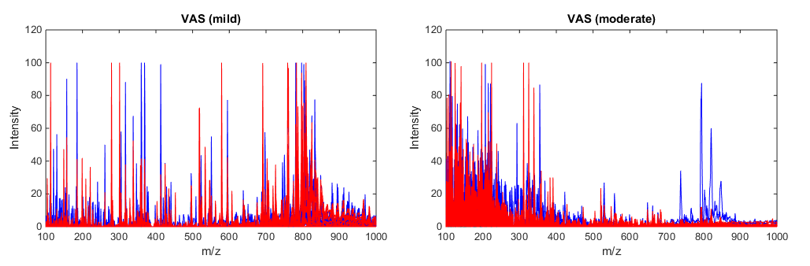
**

**Figure S1a:** Mass spectra for the VAS symptom, with application of baseline and ROI correction, with samples from the control group (blue) and samples from the fibromyalgia group (red) obtained by PSI-MS.


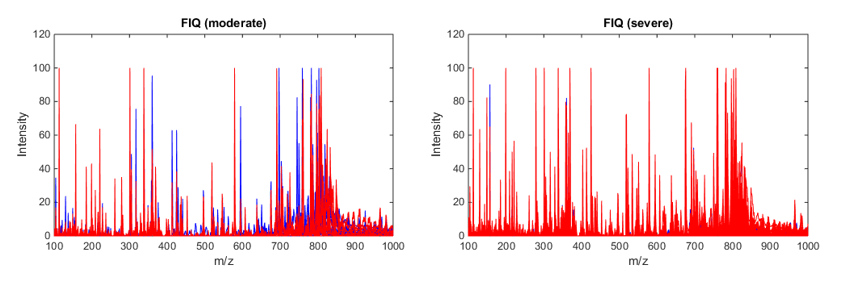


**Figure S1b:** Mass spectra for the FIQ symptom, with application of baseline and ROI correction, with samples from the control group (blue) and samples from the fibromyalgia group (red) obtained by PSI-MS.


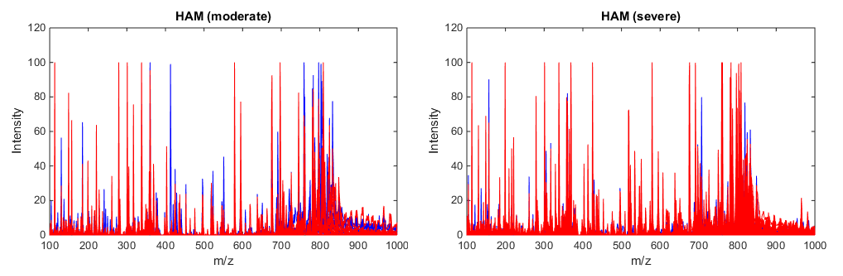


**Figure S1c:** Mass spectra for the HAM symptom, with application of baseline and ROI correction, with samples from the control group (blue) and samples from the fibromyalgia group (red) obtained by PSI-MS.

**
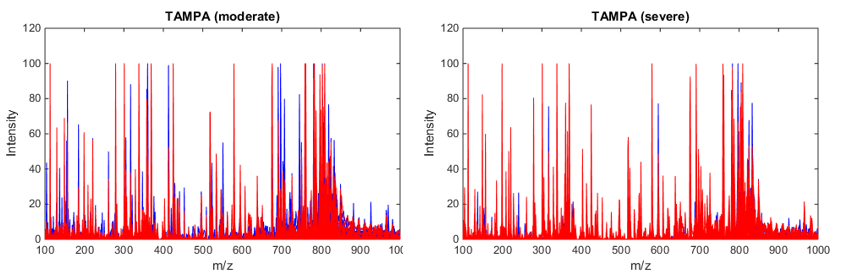
**

**Figure S1d:** Mass spectra for the TAMPA symptom, with application of baseline and ROI correction, with samples from the control group (blue) and samples from the fibromyalgia group (red) obtained by PSI-MS.


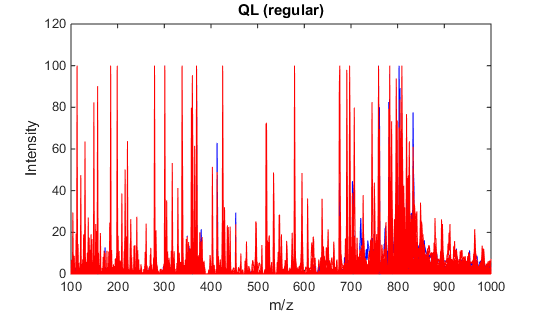


**Figure S1e:** Mass spectrum for the QL symptom, with application of baseline and ROI correction, with samples from the control group (blue) and samples from the fibromyalgia group (red) obtained by PSI-MS.

**
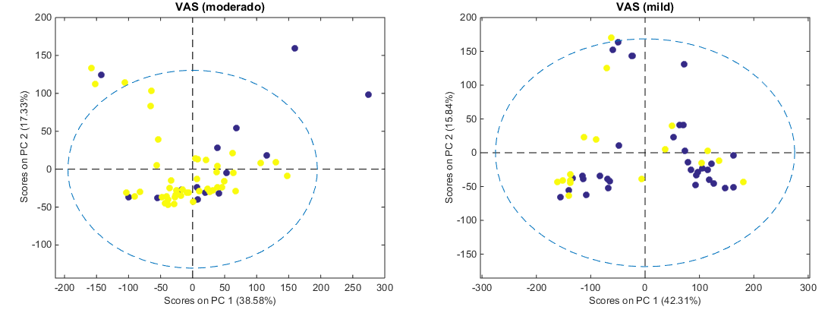
**

**Figure S2a:** Graphs of PC1 versus PC2 scores for the VAS symptom, with CG (yellow) and FG (blue) samples and confidence ellipses (dashed circles) for the analyzed datasets. The percentage of total variance for each PC is described in parentheses.


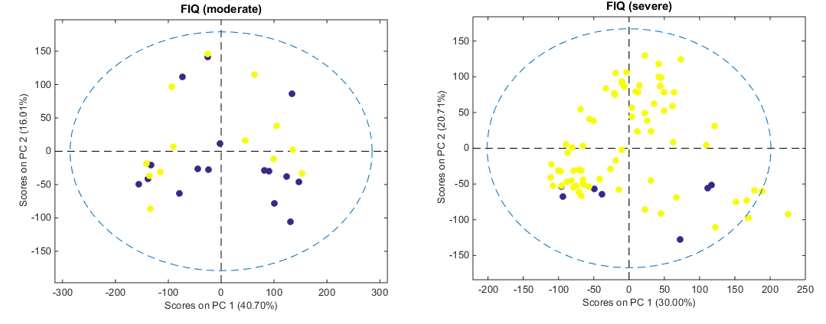


**Figure S2b:** Graphs of PC1 versus PC2 scores for the FIQ symptom, with CG (yellow) and FG (blue) samples and confidence ellipses (dashed circles) for the analyzed datasets. The percentage of total variance for each PC is described in parentheses.


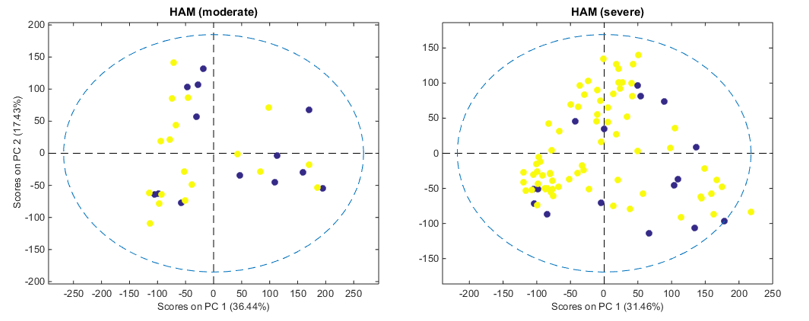


**Figure S2c:** Graphs of PC1 versus PC2 scores for the HAM symptom, with CG (yellow) and FG (blue) samples and confidence ellipses (dashed circles) for the analyzed datasets. The percentage of total variance for each PC is described in parentheses.


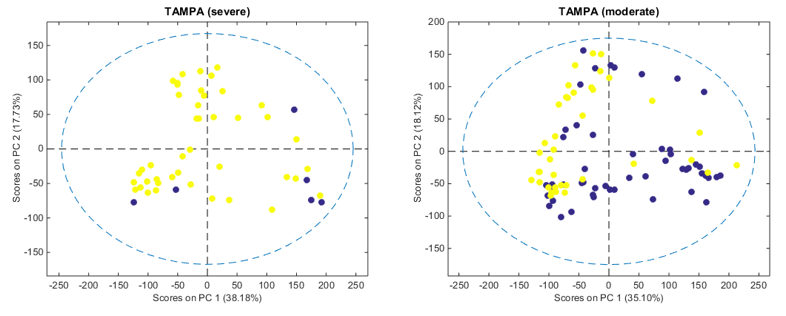


**Figure S2d:** Graphs of PC1 versus PC2 scores for the TAMPA symptom, with CG (yellow) and FG (blue) samples and confidence ellipses (dashed circles) for the analyzed datasets. The percentage of total variance for each PC is described in parentheses.


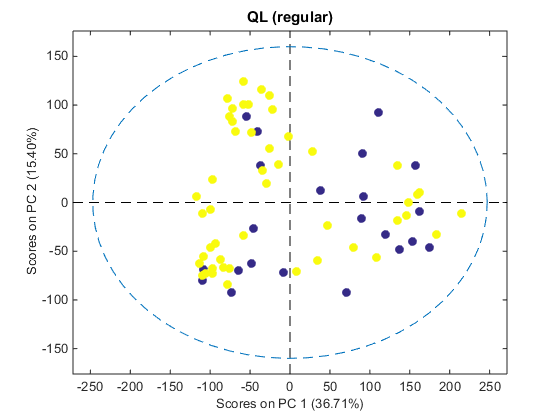


**Figure S2e:** Graphic of PC1 versus PC2 scores for the QL symptom, with CG (yellow) and FG (blue) samples and confidence ellipses (dashed circles) for the analyzed datasets. The percentage of total variance for each PC is described in parentheses.

| Symptom | Levels | Number of patients Without  FM | Number of patients With  FM | Symptom | Levels | Number of patients Without  FM | Number of patients With  FM |
| --- | --- | --- | --- | --- | --- | --- | --- |
| **VAS** | no pain | 31 | 2 | **QL** | low | 3 | 37 |
|  | mild | 33 | 16 |  | regular | 22 | 48 |
|  | moderate | 14 | 47 |  | good | 34 | 0 |
|  | severe | 0 | 18 |  | Excellent | 19 | 0 |
| **FIQ** | mild | 59 | 0 | **TAMPA** | mild | 19 | 2 |
|  | moderate | 15 | 13 |  | moderate | 52 | 38 |
|  | severe | 7 | 73 |  | severe | 7 | 43 |
| **HAM** | mild | 44 | 1 | **CAT** | mild | 48 | 10 |
|  | moderate | 19 | 13 |  | moderate | 27 | 37 |
|  | severe | 15 | 71 |  | severe | 3 | 33 |

**Table S1:** sample arrangement and symptom levels, with the respective numbers of plasma samples analyzed.

| Analyzed data sets | | | |
| --- | --- | --- | --- |
| Symptom | level | Control group | Case group |
| VAS | mild | 33 | 16 |
|  | moderate | 14 | 47 |
|  | mild + moderate | 47 | 63 |
| FIQ | moderate | 15 | 13 |
|  | severe | 7 | 73 |
|  | moderate + severe | 22 | 86 |
| HAM | moderate | 19 | 13 |
|  | severe | 15 | 71 |
|  | moderate + severe | 34 | 84 |
| QL | regular | 22 | 48 |
| TAMPA | moderate | 52 | 38 |
|  | severe | 7 | 43 |
|  | moderate + severe | 59 | 81 |
| CAT | mild | 48 | 10 |
|  | moderate | 27 | 37 |
|  | mild + moderate | 75 | 47 |

**Table S2:** Dataset analyzed in this study, with the respective numbers of samples from the control and the case group (fibromyalgia).


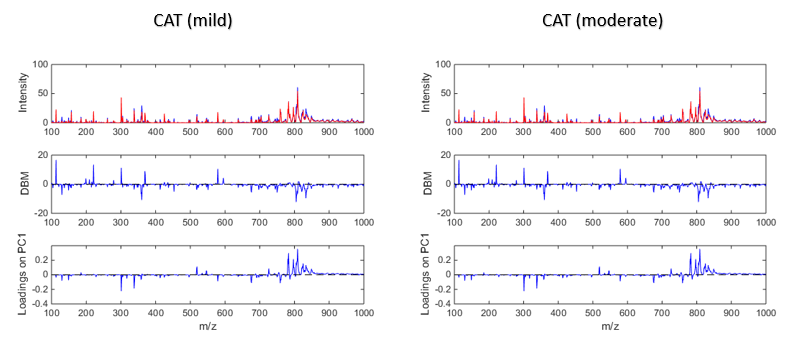
**Figure S3a:** Spectral average with all samples (CG and FG groups) for the CAT symptom, followed by the difference in spectral averages between the two groups (DBM) and PC1 loadings.


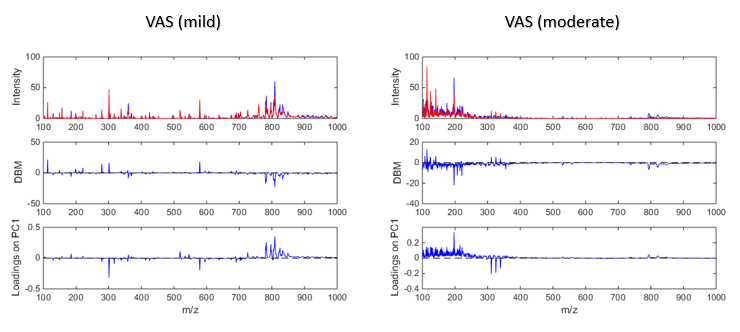


**Figure S3b:** Spectral average with all samples (CG and FG groups) for the VAS symptom, followed by the difference in spectral averages between the two groups (DBM) and PC1 loadings.


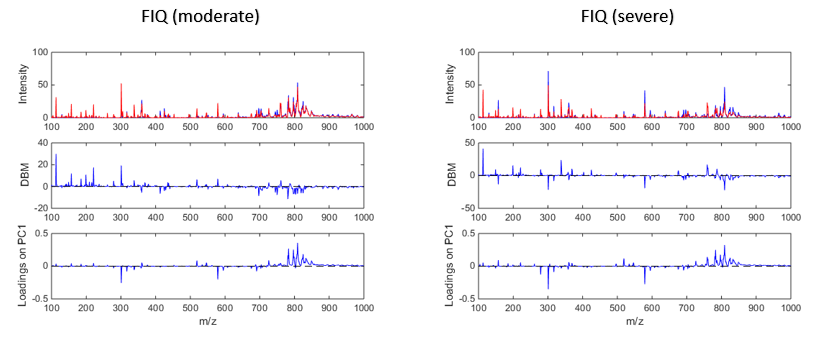
**Figure S3c:** Spectral average with all samples (CG and FG groups) for the FIQ symptom, followed by the difference in spectral averages between the two groups (DBM) and PC1 loadings.


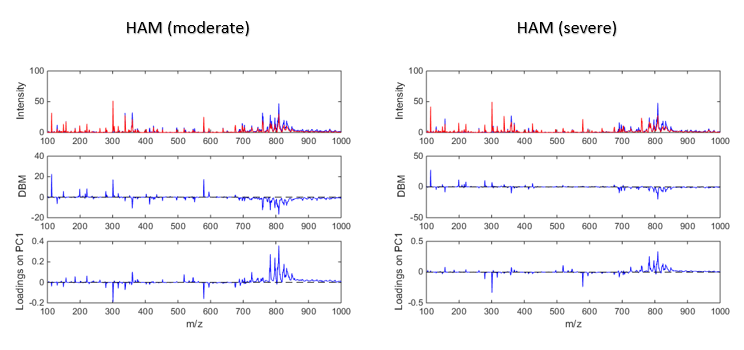


**Figure S3d:** Spectral average with all samples (CG and FG groups) for the HAM symptom, followed by the difference in spectral averages between the two groups (DBM) and PC1 loadings.


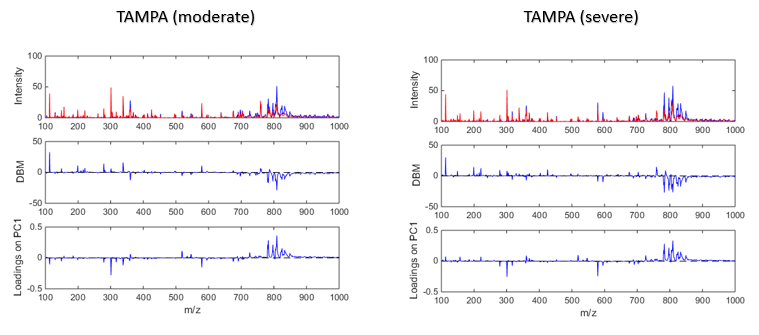
**Figure S3e:** Spectral average with all samples (CG and FG groups) for the TAMPA symptom, followed by the difference in spectral averages between the two groups (DBM) and PC1 loadings.


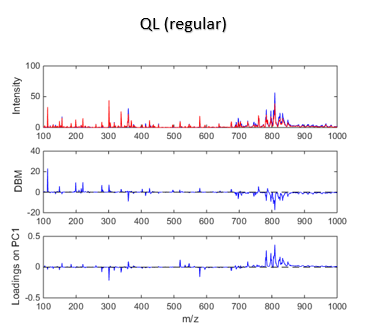


**Figure S3f:** Spectral average with all samples (CG and FG groups) for the QL symptom, followed by the difference in spectral averages between the two groups (DBM) and PC1 loadings.

| Variable  (m/z) | Main class | Subclass | Common name | Formula |
| --- | --- | --- | --- | --- |
| 645 | Glycerolipids | Diradylglycerols | DG(16:0/22:4(7Z,10Z,13Z,16Z)/0:0) | C_41_H_72_O_5_ |
| 679 | Steroids and derivatives | Steroid esters | CE(20:1(11Z)) | C_47_H_82_O_2_ |
| 707 | Steroids and derivatives | Steroid esters | CE(22:1(13Z)) | C_49_H_86_O_2_ |
| 797 | Glycerophospholipids | Glycerophosphoglycerols | PG(18:2(9Z,12Z)/20:3(5Z,8Z,11Z)) | C_44_H_77_O_10_P |
|  |  |  | PG(16:0/22:5(7Z,10Z,13Z,16Z,19Z)) | C_44_H_77_O_10_P |
|  |  |  | PG(16:1(9Z)/22:4(7Z,10Z,13Z,16Z)) | C_44_H_77_O_10_P |
|  |  |  | [PG(18:1(11Z)/20:4(5Z,8Z,11Z,14Z))](https://hmdb.ca/metabolites/HMDB0010625) | C_44_H_77_O_10_P |
|  |  |  | [PG(18:1(9Z)/20:4(5Z,8Z,11Z,14Z))](https://hmdb.ca/metabolites/HMDB0010640) | C_44_H_77_O_10_P |
|  |  |  | [PG(18:2(9Z,12Z)/20:3(5Z,8Z,11Z))](https://hmdb.ca/metabolites/HMDB0010653) | C_44_H_77_O_10_P |
|  |  |  | [PG(18:2(9Z,12Z)/20:3(8Z,11Z,14Z))](https://hmdb.ca/metabolites/HMDB0010654) | C_44_H_77_O_10_P |
| 827 | Glycerolipids | Triradylcglycerols | TG(16:1(9Z)/16:1(9Z)/18:2(9Z,12Z)) | C_53_H_94_O_6_ |
|  |  |  | [TG(16:1(9Z)/16:0/18:3(9Z,12Z,15Z))](https://hmdb.ca/metabolites/HMDB0010424) | C_53_H_94_O_6_ |
|  |  |  | TG(18:2(9Z,12Z)/14:0/18:2(9Z,12Z)) | C_53_H_94_O_6_ |
|  | Sphingolipids | Phosphosphingolipids | SM(d19:1/24:1(15Z)) | C_48_H_95_N_2_O_6_P |
|  | Glycerophospholipids | Glycerophosphoglycerols | PG(18:0/22:4(7Z,10Z,13Z,16Z)) | C_46_H_83_O_10_P |
| 841 | Sphingolipids | Phosphosphingolipids | SM(d18:1/26:1(17Z)) | C_49_H_97_N_2_O_6_P |
| 848 | Glycerophospholipids | Glycerophosphoserines | PS(P-20:0/22:6(4Z,7Z,10Z,13Z,16Z,19Z)) | C_48_H_82_NO_9_P |

**Table S3:** information extracted from the HMDB database with the variables selected by the GA models used in this study.

| Variable (m/z) | Main class | Common name | Formula |
| --- | --- | --- | --- |
| 131 | Fatty Acids and Conjugates [FA01] | cis-Glutaconic acid | C_5_H_6_O_4_ |
|  |  | Citraconic acid | C_5_H_6_O_4_ |
|  |  | Glutaconic acid | C_5_H_6_O_4_ |
|  |  | Itaconic acid | C_5_H_6_O_4_ |
|  |  | Mesaconic acid | C_5_H_6_O_4_ |
|  | Fatty esters [FA07] | Methyl hydrogen fumarate | C_5_H_6_O_4_ |
|  | Fatty Acids and Conjugates [FA01] | 2oxo-3R-methyl-pentanoic acid | C_6_H_10_O_3_ |
|  |  | 3-oxo-4-methyl-pentanoic acid | C_6_H_10_O_3_ |
|  |  | 3S-methyl-2-oxo-pentanoic acid | C_6_H_10_O_3_ |
|  |  | 2-keto-n-caproic acid | C_6_H_10_O_3_ |
|  |  | 3-keto-n-caproic acid | C_6_H_10_O_3_ |
|  |  | 4-keto-n-caproic acid | C_6_H_10_O_3_ |
|  |  | 5-keto-n-caproic acid | C_6_H_10_O_3_ |
|  | Fatty esters [FA07] | 4-Hydroxy-4-methyltetrahydro-2H-pyran-2-one | C_6_H_10_O_3_ |
| 133 | Fatty Acids and Conjugates [FA01] | 2-hydroxy-2-butenedioic acid | C_4_H_4_O_5_ |
|  |  | Oxalacetic acid | C_4_H_4_O_5_ |
|  |  | 2,4,6-Octatriynoic acid | C_8_H_4_O_2_ |
|  |  | 3-hydroxy-3-methyl-2-oxo-butanoic acid | C_5_H_8_O_4_ |
|  |  | 2-Acetolactate | C_5_H_8_O_4_ |
|  |  | 2-Hydroxy-4-oxopentanoic acid | C_5_H_8_O_4_ |
|  |  | (S)-2-Acetolactate | C_5_H_8_O_4_ |
|  |  | (S)-4-hydroxy-2-oxopentanoic acid | C_5_H_8_O_4_ |
|  |  | dimethyl-malonic acid | C_5_H_8_O_4_ |
|  |  | Ethylmalonic acid | C_5_H_8_O_4_ |
|  |  | Methylsuccinic acid | C_5_H_8_O_4_ |
|  |  | L-Isoleucic acid | C_6_H_12_O_3_ |
|  |  | 2-ethyl-2-hydroxy-butyric acid | C_6_H_12_O_3_ |
|  |  | 2-hydroxy-3-methyl-pentanoic acid | C_6_H_12_O_3_ |
|  |  | (2R,3S)-2-hydroxy-3-methylpentanoic acid | C_6_H_12_O_3_ |
|  |  | (2S,3R)-3-hydroxy-2-methylpentanoic acid | C_6_H_12_O_3_ |
|  |  | 3R-hydroxy-hexanoic acid | C_6_H_12_O_3_ |
|  |  | 5-hydroxy caproic acid | C_6_H_12_O_3_ |
|  |  | 5R-hydroxy-hexanoic acid | C_6_H_12_O_3_ |
|  |  | 6-hydroxy caproic acid | C_6_H_12_O_3_ |
|  |  | DL-2-hydroxy caproic acid | C_6_H_12_O_3_ |
|  |  | DL-3-hydroxy caproic acid | C_6_H_12_O_3_ |
|  |  | DL-4-hydroxy caproic acid | C_6_H_12_O_3_ |
|  |  | D-Leucic acid | C_6_H_12_O_3_ |
|  |  | hydroxy-isocaproic acid | C_6_H_12_O_3_ |
|  |  | 5R-hydroxy-hexanoic acid | C_6_H_12_O_3_ |
|  |  | Leucinic acid | C_6_H_12_O_3_ |
|  |  | (S)-3-hydroxyhexanoic acid | C_6_H_12_O_3_ |
| 255 | Fatty Acids and Conjugates [FA01] | 4-methyl-5-carboxyethyl-2-furanacrylic acid | C_11_H_12_O_5_ |

**Table S4:** information extracted from the LIPID MAPS database with the variables selected by the GA models used in this study.
